# Editorial: Wnt Signaling in Immune Cell Regulation During Microbial Infection and Cancer

**DOI:** 10.3389/fimmu.2020.01133

**Published:** 2020-06-05

**Authors:** Antje Blumenthal, Elena Martin-Orozco, Jere W. McBride, Dennis A. Carson, Malini Sen

**Affiliations:** ^1^The University of Queensland Diamantina Institute, The University of Queensland, Brisbane, QLD, Australia; ^2^Department of Biochemistry and Molecular Biology B and Immunology, Murcia BioHealth Research Institute (IMIB), University of Murcia, Murcia, Spain; ^3^Departments of Pathology and Microbiology and Immunology, Center for Biodefense and Emerging Infectious Diseases, University of Texas Medical Branch at Galveston, Galveston, TX, United States; ^4^Department of Medicine, University of California, San Diego, San Diego, CA, United States; ^5^Cancer Biology and Inflammatory Disorder, Indian Institute of Chemical Biology (CSIR), Kolkata, India

**Keywords:** WNT, immunity, infection, microbe, pathogen, tumor

## Overview

WNT ligands interact with distinct families of cell surface receptors to initiate signaling in response to the various environmental cues that orchestrate cell physiology and tissue homeostasis. For example, WNT signaling directs cytoskeletal modulations, organelle dynamics and specific transcriptional programs that regulate cell growth, differentiation, and migration. While WNT signaling was initially best studied in embryogenesis and carcinogenesis, quite naturally, this important aspect of life was also discovered to orchestrate interactions between the immune system and invading pathogens and tumors, as well as the microbiota that co-exist with the host. Many gaps remain in our understanding of how WNT signaling is involved in immune defense, host cell interactions with tumors and phylogenetically diverse microbes. This Research Topic comprises articles that provide a comprehensive picture of the current understanding of WNT signaling in response to pathogens, commensals, and tumors. With a view toward exploiting these novel insights for therapeutic applications, considerations of targeting specific WNTs and WNT signaling intermediates in the various scenarios are discussed.

## WNT Signaling Scheme

WNTs comprise a family of secreted glycol-lipo-protein ligands that bind to cell surface receptors including Frizzled G protein-coupled receptors, as well as ROR and Ryk tyrosine kinases to elicit signal transduction. Although WNT signaling is broadly categorized into 2 principal types, β-catenin-dependent and β-catenin independent, considerable crosstalk exists among the signaling intermediates of WNT signaling pathways in complex modes that are mostly context dependent. The intricate features of the diverse WNT signaling cascades under physiological states and different pathophysiological settings have been covered categorically in the articles by Mukherjee et al., Ljungberg et al., Silva-Garcia et al., Jati et al., Rogan et al., Li et al., Cosin-Roger et al., Patel et al., and  Martin-Orozco et al..

## WNT Signaling in Relation to Microbial Pathogenesis

Being an important component of cell and tissue homeostasis, WNT signaling is often exploited by pathogens for their own survival. For example, *Salmonella* Typhimurium depresses WNT signaling in the endothelium by virtue of its T3SS effector system and utilizes the vascular leakiness to enhance its dissemination (Rogan et al.). Some pathogens, for example *S. flexneri* on the other hand, take advantage of the inflammatory component of WNT signaling for propagation of infection (Mukherjee et al.). Along the same lines, Silva-Garcia et al. have outlined the role of various bacterial virulence factors in the regulation of the WNT-β-catenin pathway during disease pathogenesis. Ljungberg et al. provide an overview of bacteria-induced WNT responses in experimental systems and patient samples, and the functional consequences in the context of immune responses. Some examples include impact on immune cell differentiation, antimicrobial defense, inflammation, and the cross-talk between innate and adaptive immunity. Adding another twist to this topic, Jati et al. have described how actin cytoskeleton organization by WNT5A signaling dictates the killing of some pathogens by utilizing the host autophagy machinery. Jati et al. also raise how WNT signaling might facilitate differentiating between pathogenic and non-pathogenic microbes, given the plethora of beneficial commensals that have evolved with our system.

In view of the ongoing tug of war between the host and pathogens for WNT signaling components during the onset and progression of infections, it would be fair to state that appropriate and targeted inhibition or activation of WNT signaling may be beneficial for the host's ability to control pathogens. Such operations, however, would naturally vary case to case.

## WNT Signaling in Tumorigenesis and Anti-Tumor Immune Responses

Consistent with an important role in directing transcription, cytoskeletal motility and cell proliferation and differentiation, dysregulated WNT signaling is associated with the development of tumors. With the discovery that WNT signaling also shapes immune cell functions, it is increasingly appreciated that the immune response to tumors is shaped by WNT signaling in both tumor and immune cells. As explained by Cosin-Roger et al., evidence of the association of WNT signaling with tumorigenesis was first reported in 1991, when mutations in the Adenomatous Polyposis Coli (APC) gene were linked with the development of colorectal cancer. Anomalous expression and function of the APC gene product, which regulates β-catenin functions as a transcriptional co-activator, is now known to be associated with uncontrolled cell proliferation in cancer. Cosin-Roger et al. and Li et al. project WNT-β-catenin signaling as a high priority target for therapeutic intervention in the treatment of cancers. In their review, Patel et al. describe the role of tumor infiltrating macrophages in colorectal cancer, commenting on the high level of expression of WNT2 and WNT5A in the macrophages and its correlation with cancer progression. They further describe how WNT-β-catenin signaling is involved in the functional polarization of tumor-associated macrophages that facilitates tumor growth. Martin-Orozco et al. discuss the role of WNT-β-catenin signaling in augmenting drug resistance of tumors through expression of drug export pumps. Importantly, reports of the role of WNT signaling in tumor regression also exist. For example, evidence has been presented that WNT5A acts as a tumor suppressor in various cancers. In fact Foxy-5 is a hexapeptide mimic of WNT5A, which is currently being studied in clinical trials as a tumor suppressor (Patel et al.).

As dysregulated WNT signaling is explored as a therapeutic target in cancer, it is important to consider that the applied interventions will define and re-shape the associated immune response. With pro- and anti-tumorigenic functions of WNT signaling in specific tumor settings it will be important to evaluate the therapeutic benefits and potential risks of WNT-targeted interventions in different cancers.

## Perspective and Future Directions

Activation of WNT signaling is an integral part of host responses to microbial encounter and tumorigenesis. Given the complexity of WNT signaling, special emphasis should be placed on the nature of the WNT response, as well as the understanding of functional impact of individual WNT ligands and their concerted action in response to infection and tumorigenesis. This will be essential to evaluate strategies for WNT-directed therapeutic interventions that might prove particularly valuable considering the significant challenges posed by drug resistance in pathogens as well as tumors.

## Author Contributions

All authors listed have made a substantial, direct and intellectual contribution to the work, and approved it for publication.

## Conflict of Interest

The authors declare that the research was conducted in the absence of any commercial or financial relationships that could be construed as a potential conflict of interest.

